# Mechanisms of Wharton’s Jelly-derived MSCs in enhancing peripheral nerve regeneration

**DOI:** 10.1038/s41598-023-48495-6

**Published:** 2023-12-01

**Authors:** Young Ho Shin, Soon Jin Choi, Jae Kwang Kim

**Affiliations:** 1 Department of Orthopedic Surgery, Asan Medical Center, University of Ulsan College of Medicine, 88, Olympic Road 43‐gil, Songpa‐gu, Seoul, 05505 South Korea; 2 Asan Institute for Life Sciences, Seoul, Korea

**Keywords:** Regenerative medicine, Mesenchymal stem cells

## Abstract

Warton’s jelly-derived Mesenchymal stem cells (WJ-MSCs) play key roles in improving nerve regeneration in acellular nerve grafts (ANGs); however, the mechanism of WJ-MSCs-related nerve regeneration remains unclear. This study investigated how WJ-MSCs contribute to peripheral nerve regeneration by examining immunomodulatory and paracrine effects, and differentiation potential. To this end, WJ-MSCs were isolated from umbilical cords, and ANGs (control) or WJ-MSCs-loaded ANGs (WJ-MSCs group) were transplanted in injury animal model. Functional recovery was evaluated by ankle angle and tetanic force measurements up to 16 weeks post-surgery. Tissue biopsies at 3, 7, and 14 days post-transplantation were used to analyze macrophage markers and interleukin (IL) levels, paracrine effects, and MSC differentiation potential by quantitative real-time polymerase chain reaction (RT-qPCR) and immunofluorescence staining. The WJ-MSCs group showed significantly higher ankle angle at 4 weeks and higher isometric tetanic force at 16 weeks, and increased expression of CD206 and IL10 at 7 or 14 days than the control group. Increased levels of neurotrophic and vascular growth factors were observed at 14 days. The WJ-MSCs group showed higher expression levels of S100β; however, the co-staining of human nuclei was faint. This study demonstrates that WJ-MSCs' immunomodulation and paracrine actions contribute to peripheral nerve regeneration more than their differentiation potential.

## Introduction

Autologous nerve graft is the standard treatment option for peripheral nerve defect reconstruction; however, donor-side morbidities, limited donor tissue length, and donor–recipient size mismatch limit its use^1,2^. Therefore, alternative materials were introduced. Among them, acellular nerve grafts (ANGs) have advantages in retaining the microstructure of extracellular matrix (ECM) proteins^2,3^. However, ANGs alone have limited regeneration potential, especially in large nerve defects, which originates from the lack of neuronal support cells including Schwann cells (SCs)^4,5^.

Previous studies revealed the efficacy of SCs for enhancing nerve regeneration after nerve graft^4,6^. However, the clinical application of SCs to nerve graft is limited by their limited resources and technically challenging culture process^5,7^. To overcome these limitations, mesenchymal stem cells (MSCs), which are a kind of precursor cells to SCs, are being considered as an alternative^8^.

MSCs are self-renewal multipotent precursor cells derived from various tissues (e.g., bone marrows, adipose tissues, skin, and umbilical cords)^9^. Bone marrows-MSCs (BM-MSCs) are the most widely investigated type of MSCs; however, their low yield and high degree of invasiveness have limited their clinical applicability^10^. Adipose tissue-derived MSCs (AMSCs) have several advantages over BMSCs, including lower degree of invasiveness for acquisition, and rapid rate of expansion in vitro; however, the proliferation rate and differentiation potential of these cells decrease with increasing donor age^11,12^. Among various MSCs, WJ-MSCs, which are isolated from human umbilical cords as waste tissue, have multiple advantages, including a non-invasive harvesting method, high proliferation rate, and high expression of pluripotency markers^5,13,14^.

One previous study revealed that WJ-MSCs could differentiate to SC-like cells (SCLCs) to aid nerve regeneration in peripheral injury animal model^5^. However, research on the mechanism of MSCs related to peripheral nerve regeneration is insufficient, and the mechanism of MSCs differentiation and secretion of neurotransmitters in peripheral nerves are yet to be clarified^15^. In the present study, the mechanisms involved in peripheral nerve regeneration by WJ-MSCs were investigated, focusing on three perspectives: immunomodulatory effects, paracrine effects, and differentiation potential.

## Results

### Functional assessment and analysis of toluidine blue staining

**Figure 1:**
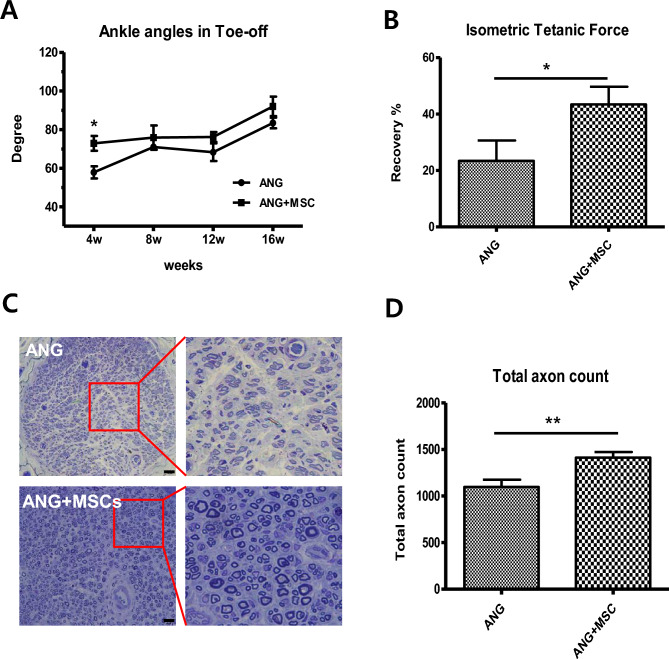
In vivo functional recovery. (**A**) Video analysis of gait angles at the toe-off phase. The angles were measured in the control and WJ-MSCs groups for up to 16 weeks following transplantation surgery. (**B**) Measurement of the isometric tetanic force at 16 weeks postoperatively. (**C**) Microscopic examination of toluidine blue-stained distal sciatic nerve ends at 16 weeks postoperatively showing the histological structures of nerve fiber, axon, and myelin. Scale bar = 20 μm. (**D**) Total axon count is shown. The mean ± SEM values of the control and WJ-MSCs groups are compared (*p < 0.05, **p < 0.01, Student’s t-test).

The ankle angles at the toe‐off phase at 4, 8, 12, and 16 weeks were 57.8° ± 3.1°, 71.0° ± 1.67°, 68.2° ± 4.5°, and 83.4° ± 2.7° for the ANG group, and 72.8° ± 3.8°, 75.8° ± 6.3°, 76.1° ± 2.5°, and 92.0° ± 5.0° for the ANG + WJ-MSCs group (Fig. 1A). Although the WJ-MSCs group exhibited higher ankle angles than the ANG group, statistical significance was observed at 4 weeks postoperatively (*p* = 0.010). Measurement of the isometric tetanic force at 16 weeks postoperatively revealed that the ANG + WJ-MSCs group exhibited a statistically significant recovery rate than the ANG group (43.4 ± 6.2% vs. 23.4 ± 7.1%, *p* = 0.029; Fig. 1B). Toluidine Blue staining was employed to observe the histological structures of axons and nerve fibers (Fig. 1C). Results revealed that the total axon counts were significantly higher in the ANG + WJ-MSCs group than in the ANG group (1412.0 ± 60.7 vs. 1098.0 ± 78.2, *p* = 0.003; Fig. 1D).

### Expression of macrophage markers

**Figure 2:**
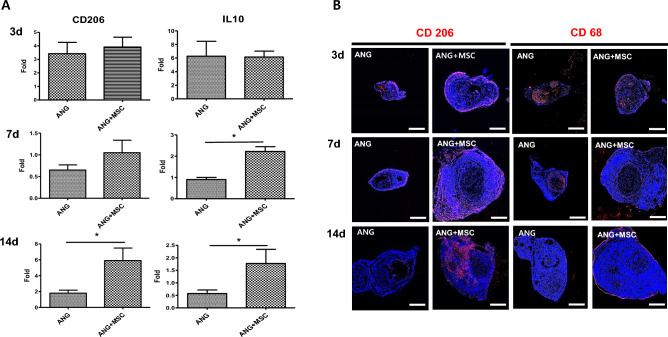
Analysis of the expression of macrophage markers. (**A**) RT-qPCR analysis of CD206 and IL10 in the control and WJ-MSCs groups. Expression levels were normalized against GAPDH expression. (**B**) Immunofluorescence for CD206 and CD68 in cross-sections at 3, 7, and 14 days post-implantation as indicated. Scale bar = 500 μm (*p < 0.05, Student’s t-test).

The mRNA expression levels of CD206 and IL10 were not significantly different between the two groups at 3 days postoperatively. The mRNA expression level of CD206 was significantly higher in the WJ-MSCs group than in the control group at 14 days postoperatively (*p* < 0.05). Meanwhile, the mRNA expression level of IL10 was significantly higher in the WJ-MSCs group than in the control group at 7 and 14 days postoperatively (*p* < 0.05) (Fig. 2A). In the analysis of protein expression levels, CD206 expression was maintained at a higher level in the WJ -MSCs group than in the control group until 14 days postoperatively. However, CD68 expression was not different between the two groups (Fig. 2B).

### Expression of neurotrophic and angiogenesis factors

**Figure 3:**
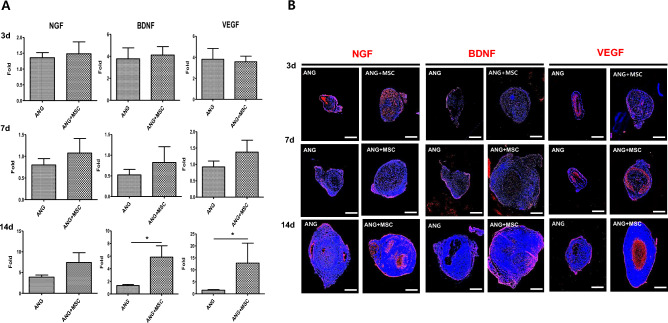
Analysis of the expression of angiogenesis and neurotrophic factors. (**A**) RT-qPCR analysis of NGF, BDNF, and VEGF in the control and WJ-MSCs groups. Expression levels were normalized against GAPDH expression. (**B**) Immunofluorescence for NGF, BDNF, and VEGF in cross-sections at 3, 7, and 14 days post-transplantation as indicated. Scale bar = 500 μm (*p < 0.05, Student’s t-test).

The mRNA expression levels of the nerve growth factor (NGF), brain-derived neurotrophic factor (BDNF), and vascular endothelial growth factors (VEGF) were not significantly different between the two groups at 3 and 7 days postoperatively. However, the mRNA expression levels of BDNF and VEGF were significantly higher in the WJ-MSCs group than in the control group at 14 days postoperatively (*p* < 0.05) (Fig. 3A). In addition, immunofluorescence staining confirmed that the protein levels of NGF, BDNF, and VEGF were higher in the WJ-MSCs group than in the control group, showing a similar pattern to RNA expression (Fig. 3B).

### Expression of SC markers and results of double-staining with human nuclei and S100β

**Figure 4:**
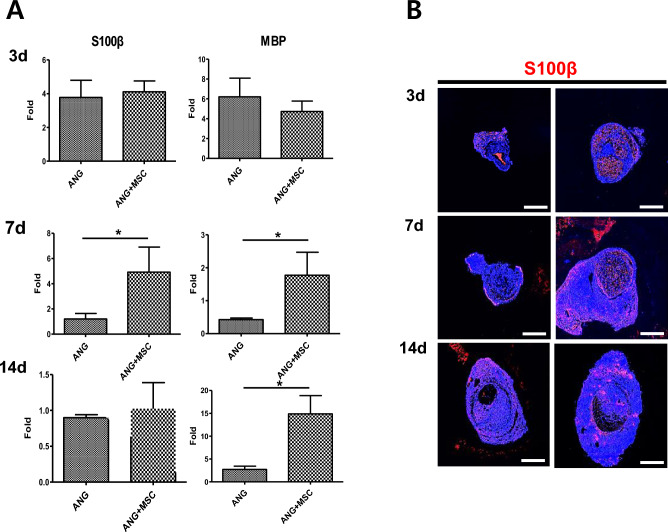
Analysis of the expression of SC markers. (**A**) RT-qPCR analysis of S100b and MBP in the control and WJ-MSCs groups. Expression levels were normalized against GAPDH expression. (**B**) Immunofluorescence for S100β in cross-sections at 3, 7, and 14 days post-transplantation as indicated. Scale bar = 500 μm (*p < 0.05, Student’s t-test).

**Figure 5:**
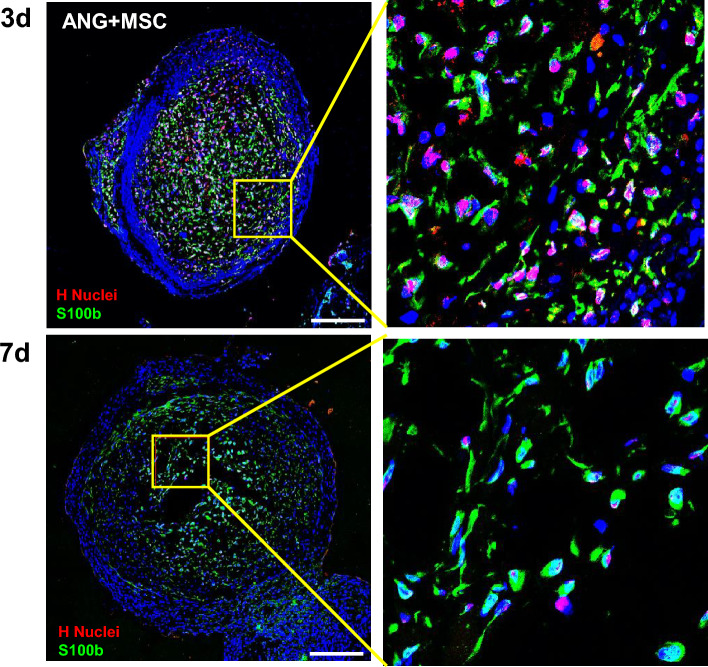
Analysis of double-staining of S100β and human nuclei. Immunofluorescence co-staining of human nuclei and S100β in cross-sections of the control and WJ-MSCs groups at 3 and 7 days. Scale bar = 200 μm.

The mRNA expression levels of S100β and myelin basic protein (MBP) were not significantly different between the two groups at 3 days postoperatively. However, they were significantly higher in the WJ-MSCs group than in the control group at 7 days postoperatively (*p* < 0.05). This difference was maintained until 14 days postoperatively only for MBP (*p* < 0.05). In addition, immunofluorescence staining showed similar results (Fig. 4). Double-staining of human nuclei and S100β in the WJ-MSCs group showed that the red-stained human nuclei and green-stained S100β did not co-colonize at 7 days postoperatively (Fig. 5).

## Discussion

Over the years, MSCs have attracted considerable interest in regenerative medicine; they are being considered in in vitro and in vivo studies to test their efficacy in enhancing peripheral nerve regeneration^16^. Additionally, they are believed to replace injured tissue cells via differentiation and maximize the intrinsic regenerative capacity of injured tissues by producing several growth factors and cytokines^17^. The mechanisms of MSCs for enhancing nerve regeneration depend on their type; however, no in-depth studies have evaluated these mechanisms in WJ-MSCs^18^.

After peripheral nerve injury, SCs trigger a series of immunoregulatory reactions by secreting cytokines and recruiting various immune cells^19^. In this process, MSCs can produce various immunoregulatory factors that modulate immune function (immunomodulatory effects)^20^. This phenomenon has been revealed in several types of MSCs^21^, but not in WJ-MSCs. Generally, macrophages can be divided into two groups: M1 macrophages, which stimulate local inflammation and remove debris at the injury site, and M2 macrophages, which play an important role in wound healing and tissue repair^22^. In this study, the WJ-MSCs group showed significantly higher level of macrophage markers, including CD206, which is a marker for an anti-inflammatory M2 macrophage^23^ and IL10, which is known for its anti-inflammatory properties^24^. However, the protein expression level of CD68, as a pan-macrophage marker that reflects macrophage quantities of M1 and M2^25,26^, was not significantly different between the two groups. This finding indicates that the number of recruited macrophages was not significantly different. The roles of M1 and M2 macrophages are nuanced and flexible^27^. Pro-inflammatory macrophages can even convert to anti-inflammatory phenotypes, demonstrating their remarkable plasticity^28^. WJ-MSCs can enhance the polarization of macrophages toward the M2 phenotype, promoting an anti-inflammatory environment^29^. Anti-inflammatory factors have several beneficial effects on nerve regeneration, including activating and proliferating SCs^30^, promoting angiogenesis^31^, and modulating the ECM environment to favor axonal growth^32^.

It is known that MSCs secrete various factors that promote nerve cell survival and axonal growth, showing increased paracrine effects^18,33^. In this study, the WJ-MSCs group showed significantly higher levels of neurotrophic (BDNF and NGF) and angiogenesis factors (VEGF). Additionally, BDNF is a neurotrophic factor that supports neuronal survival, growth, and differentiation^34^. After peripheral nerve injury, BDNF is upregulated and sustained over the course of weeks^35,36^. It promotes axonal growth, provides essential cues for axonal sprouting, and directs axonal regeneration toward their target, facilitating functional recovery^37,38^. Similar to BDNF, NGF stimulate neuronal survival, promote axonal growth, and exerts a neuroprotective effect against nerve system disorders^39,40^. Further, VEGF is a potent angiogenic factor that plays a critical role in angiogenesis, which is essential for the revascularization of regenerating nerves^41^. It exhibits direct neuroprotective effects on neurons^42^ and enhances SC proliferation and migration after peripheral nerve injury^43^. In addition to BDNF and VEGF, WJ-MSCs secrete various growth factors and cytokines that collectively contribute to paracrine effects and promote nerve regeneration. These factors include NGF, fibroblast growth factor, and insulin-like growth factor^18^.

In this study, the WJ-MSCs group exhibited higher levels of SCs markers than the control group. To determine the reason for the higher levels of SCs markers (S100β) in the WJ-MSC group, we performed the double staining of human nuclei and the S100β antibodies. If the transplanted WJ-MSCs were differentiated into Schwann cells, the expression of S100β and h nuclei, as a specific marker of human cells, would have co-localized. However, double-staining of human nuclei and S100β in the WJ-MSCs group did not show co-colonization, suggesting that the observed increase in the SC markers may be attributed to the recruitment of host SCs rather than the direct differentiation of WJ-MSCs into SCs. WJ-MSCs can differentiate into SCLCs in vitro, which promote peripheral nerve regeneration in ANGs in vivo^5^. However, the differentiation of MSCs into SCLCs is a time-consuming process and typically requires the use of specific growth factors and signaling molecules^44^. To enhance peripheral nerve regeneration by utilizing the differentiation potential of MSCs, the in vitro application of differentiated MSCs may be more beneficial than using naïve MSCs^7^. Further studies are needed to confirm whether the benefits obtained from the direct differentiation of MSCs, when applied after differentiation, are greater than those obtained through the immunomodulatory and paracrine effects of naïve MSCs in peripheral nerve regeneration.

This study has several limitations. First, although we observed elevated levels of several markers in the WJ-MSCs group compared to the control group, we did not assess the specific biological role of each marker. Further experimental validation is necessary to confirm their effectiveness. Second, we were unable to discover new target gene and related to pathways using RNA-Seq. Discovering new marker and signaling, knocking out or overexpressing them, and proving their relationship with peripheral nerve regeneration will lead to more innovative research.

In conclusion, the present study demonstrates that the mechanism of WJ-MSCs in enhancing peripheral nerve regeneration is because of their immunomodulatory and paracrine effects rather than their differentiation potential. This finding will be useful for understanding the mechanism of MSCs for enhancing peripheral nerve regeneration.

## Materials and methods

### Preparation and culture of human WJ-MSCs

This study was approved by the Institutional Review Board of Asan Medical Center (No. 2015–0303), and the WJ-MSCs were provided by the Stem Cell Center, Asan Institute for Life Sciences, Seoul, Korea. All experiments were performed in accordance with relevant guidelines and regulations. Informed consent from the mothers was obtained for the use of umbilical cords. Umbilical cords were cut into 0.3–1.0 cm pieces without blood vessels. The matrix was minced and transferred to culture dishes in minimal essential medium supplemented with 10% fetal bovine serum and an antibiotic–antimycotic mixture at 37 °C in a 5% CO_2_ incubator in vitro as described previously^45^. When the cells reached 80% confluency, they were replated at a 1:3 split ratio.

### Preparation of ANGs

This study complied with the Animal Research: Reporting of In Vivo Experiments (ARRIVE) guidelines. All animal care and experimental procedures were approved by the Institutional Animal Care and Use Committee of Asan Medical Center and Ulsan University College of Medicine (No. 2017-12-127), and all the following methods were performed in accordance with the relevant guidelines and regulations. After isoflurane induction, rats were euthanized by CO_2_ inhalation. Sciatic nerve segments (10 mm in length) were harvested from male Sprague–Dawley (SD) rats (7–8 weeks old, weight 250–350 g) (Orient Bio Inc., Seongnam, Korea). To prepare ANGs, sciatic nerve pieces were decellularized using a series of detergents as described by Shin et al.^2^. Briefly, the nerves were treated with detergents, including aprotinin, CHAPS, and DNase and RNase solutions. Then, the decellularized segments were washed several times with phosphate-buffered saline (PBS) to remove residual reagents and stored in PBS at 4 °C until use. All solutions were autoclaved or filter-sterilized before use.

### Surgical procedure

Seventy-four adult male SD rats were randomly assigned to two groups: WJ-MSCs group (which was implanted with WJ-MSCs-laden ANG; n = 37) and control group (which was implanted with ANG only; n = 37). After anesthetization, the left sciatic nerve of rats was exposed and transected, and 10 mm of the nerve was removed. The 10-mm piece of WJ-MSCs-laden ANG or ANG was sutured using 9‐0 nylon (Ethicon, Somerville, NY) under a microscope.

### Functional assessment

Seven rats were selected from each group, and their ankle angles at the toe‐off phase were measured at 4, 8, 12, and 16 weeks postoperatively to evaluate serial functional recovery. A walking track (length 1 m, width 10 cm, and height 10 cm) was built for this test. During the test, video was acquired with a digital camera (Canon SX730HS, Canon, Tokyo, Japan) at a distance of 1 m and calibrated to prevent optical distortion. Records were repeated until three satisfactory trials were obtained per rat. The ankle angle at the toe‐off phase was measured at maximal plantar flexion in the experimental lateral ankle joint. After the foot and leg segments were manually identified in the video frames, the ankle angles at the toe‐off phase were displayed in degrees.

All rats were anesthetized at 16 weeks postoperatively, and the maximum isometric tetanic force was measured. The sciatic nerve was fully exposed through previous operation incision, and another skin incision was made anterior to the ankle to expose and transect the tibialis anterior (TA) tendon distally. The TA tendon was connected to a force transducer using a custom clamp with the knee and ankle joints immobilized to a platform. A bipolar stimulator (Grass S88, Grass Instrument Corp, Quincy, MA) was used to generate stimulus and processed on a computer using LabVIEW software (National Instruments, Austin, TX). All contractions were performed at supramaximal voltage to ensure maximal activation of all TA motor units. The strength of muscles was standardized as a percentage of the value from the contralateral side.

### Analysis using toluidine blue staining

Sciatic nerve axonal regeneration in each group was directly examined using toluidine blue staining at 16 weeks postoperatively. The implanted sciatic nerves were harvested by including the distal sites, and 2.5% glutaraldehyde solution was used for fixation. The harvested nerves were further fixed in 1% osmium tetroxide, dehydrated in ethanol, and embedded in EPON resin (Miller-Stephenson Chemical Co., Sylmar, CA, USA). Cross-sections (1 μm thick) were stained with toluidine blue to visualize myelin with light microscopy. Digital images of nonoverlapping fields were taken at 400 × magnification using unbiased random sampling. The total number of myelinated axons was calculated using ImageJ software (National Institutes of Health, Bethesda, MD).

### Quantitative real-time polymerase chain reaction (RT-qPCR)

RT-qPCR was performed to evaluate the mRNA expression levels of factors related to peripheral nerve regeneration. First, macrophage markers (CD206 and interleukin 10 [IL10]) were assessed to investigate the immunomodulatory effects of WJ-MSCs. Second, NGF, BDNF, and VEGF were assessed to analyze the paracrine effects of WJ-MSCs on ANGs. Third, SC markers (S100β and MBP) were assessed to confirm the recruitment of SCs by WJ-MSCs in ANGs. All experiments were performed using nerve grafts harvested from five rats in each group at 3, 7, and 14 days postoperatively. Gene expressions were analyzed as described previously^5^. Total RNA was isolated from ANGs or WJ-MSCs-laden ANGs using TRIzol (Thermo Fisher Scientific). Approximately 1 μg of total RNA was used for cDNA synthesis using a first-strand cDNA synthesis kit. Quantitative estimation of mRNA expression was conducted using the ABI 7500 Fast Real-Time PCR System (Applied Biosystems/Thermo Fisher Scientific). All experiments were performed in triplicates and independently repeated more than three times. The following primers were used: CD206 (forward, TTA CTT TAA GGG GGC GTG TG; reverse, AGT TGG TTG GGG AGT GTC AG), IL10 (forward, CTC CAC CTG GCA AAC AAA AT; reverse, CTG CCT AGC CCA CAA AGA AG), NGF (forward, ACT CGG CTC CTT TGA GTT GA; reverse, CCC GTC CTA CAG AAG CAG AG), BDNF (forward, GAA GGT GAG GAA AGC AGC AC; reverse, TGC ACA GTC ATC TGG AAA GC), VEGF (forward, TGC TTC CTA GTG GGC TCT GT; reverse, CAC ACA TAC ACT CCG GCA TC), S100β (forward, GAA TTG GGG CAG AGA AAT GA; reverse, GGC TTG AGC TTC TTG GAA TG), MBP (forward, AAT GTT TCA GGG CAC CGT AG; reverse, AAA AAC CAG CCA GCT GAG AA), and GAPDH (forward, ATG GTG AAG GTC CCT GTG AAC G; reverse, CTT GCC GTG GGT AGA GTC AT). The comparative C_t_ method (2^−ΔΔCt^)^46^ was used to analyze the relative amount of gene expression.

### Immunofluorescence staining

The protein levels of CD206, CD68, NGF, BDNF, VEGF, and S100β were evaluated using immunofluorescence staining at 3, 7, and 14 days postoperatively. CD68 (a total marker of all macrophages) was analyzed to identify and quantify macrophages. In addition, double-staining for human nuclei and S100β was performed only in the WJ-MSCs group at 3 and 7 days postoperatively to investigate the differentiation potential of WJ-MSCs in ANGs. All experiments were performed using nerve grafts harvested from five rats in each group.

The grafts were snap-frozen in liquid nitrogen with frozen section compound (Leica Biosystems, Wetzlar, Germany) as described previously^47^. Samples were cut into 6-μm-thick cross-sections using a Cryo-Star HM560 freezing microtome (Thermo Fisher Scientific). After fixation, sections were permeabilized and then blocked with 10% normal goat serum. Thereafter, they were incubated overnight with the primary antibody at 4 °C. The following antibodies were used: anti-CD206 (ab64693; Abcam, Cambridge, UK), anti-CD68 (ab201340; Abcam), anti-NGF (ab6199; Abcam), anti-BDNF (ab108319; Abcam), anti-VEGF (ab1316), anti-S100b (ab52642; Abcam), and anti-hNuclei (MAB1281; Millipore, Burlington, MA, USA). Secondary antibodies, anti-rabbit Alexa 555 (A32732, Invitrogen), and anti-mouse Alexa 546 (Invitrogen A11003), were applied for 1 h at room temperature in the dark. Finally, 4′,6-diamidino-2-phenylindole was used to counterstain nuclei. Analysis of staining was performed using an LSM-810 confocal microscope (Zeiss, Oberkochen, Germany) at 100 × magnification.

### Statistical analysis

All experiments were repeated three times or more. Values are presented as mean ± standard error of the mean (SEM). Statistical significance was considered at *p* < 0.05. All statistical analyses were performed using Student’s *t*-test in IBM SPSS Statistics for Windows v. 27.0 (IBM, USA).

## Data Availability

All data analyzed during this study are available from the corresponding author on reasonable request.
